# Quantifying Selective Reporting and the Proteus Phenomenon for Multiple Datasets with Similar Bias

**DOI:** 10.1371/journal.pone.0018362

**Published:** 2011-03-29

**Authors:** Thomas Pfeiffer, Lars Bertram, John P. A. Ioannidis

**Affiliations:** 1 Program for Evolutionary Dynamics, Harvard University, Cambridge, Massachusetts, United States of America; 2 Neuropsychiatric Genetics Group, Department of Vertebrate Genomics, Max-Planck Institute for Molecular Genetics, Berlin, Germany; 3 Department of Hygiene and Epidemiology, University of Ioannina School of Medicine, Biomedical Research Institute, Foundation for Research and Technology-Hellas, Ioannina, Greece; 4 Center for Genetic Epidemiology and Modeling, ICRHPS, Tufts Medical Center and Department of Medicine, Tufts University School of Medicine, Boston, Massachusetts, United States of America; 5 Department of Epidemiology, Harvard School of Public Health, Boston, Massachusetts, United States of America; 6 Stanford Prevention Research Center, Stanford University School of Medicine, Stanford, California, United States of America; University of Modena and Reggio Emilia, Italy

## Abstract

Meta-analyses play an important role in synthesizing evidence from diverse studies and datasets that address similar questions. A major obstacle for meta-analyses arises from biases in reporting. In particular, it is speculated that findings which do not achieve formal statistical significance are less likely reported than statistically significant findings. Moreover, the patterns of bias can be complex and may also depend on the timing of the research results and their relationship with previously published work. In this paper, we present an approach that is specifically designed to analyze large-scale datasets on published results. Such datasets are currently emerging in diverse research fields, particularly in molecular medicine. We use our approach to investigate a dataset on Alzheimer's disease (AD) that covers 1167 results from case-control studies on 102 genetic markers. We observe that initial studies on a genetic marker tend to be substantially more biased than subsequent replications. The chances for initial, statistically non-significant results to be published are estimated to be about 44% (95% CI, 32% to 63%) relative to statistically significant results, while statistically non-significant replications have almost the same chance to be published as statistically significant replications (84%; 95% CI, 66% to 107%). Early replications tend to be biased against initial findings, an observation previously termed Proteus phenomenon: The chances for non-significant studies going in the same direction as the initial result are estimated to be lower than the chances for non-significant studies opposing the initial result (73%; 95% CI, 55% to 96%). Such dynamic patters in bias are difficult to capture by conventional methods, where typically simple publication bias is assumed to operate. Our approach captures and corrects for complex dynamic patterns of bias, and thereby helps generating conclusions from published results that are more robust against the presence of different coexisting types of selective reporting.

## Introduction

In many research fields, meta-analyses play an increasingly important role for synthesizing evidence from studies that have been published in the past. Since not all studies and analyses that have been undertaken are eventually published in the scientific literature, meta-analyses typically rely on incomplete samples of study outcomes. These samples might be biased, because the result of a study or of a specific analysis might influence its chances to become reported. Studies with results that achieve formal statistical significance might, for example, have increased chances to become published [1]–[5]. Biases also results from the way data and outcomes are analyzed and represented in scientific publications [6]–[9]. The resulting selective reporting bias might distort the conclusions of a meta-analysis.

Statistical methods have been developed to detect and correct for selective reporting biases [10]. These methods have limitations and may even lead to misleading inferences when applied to single meta-analyses with limited data. In particular, the frequently used funnel plots and related tests have been criticized [11], [12]. Additional complications arise from the possibility that selective reporting may depend on the position of a study in the sequence of all published studies. The first published study is often the most biased one towards an extreme result. Subsequent studies might be biased against the result of the first one; this pattern has been observed for molecular medicine publications and has been referred to as the Proteus phenomenon [13], [14]. Such complex dynamic patterns of bias are difficult to account for by conventional statistical approaches that typically assume a simple bias to operate. Aside from statistical approaches, it is very difficult to verify the existence of unpublished studies or the presence of excessive selective reporting; for alternative methodologies including surveys and experimental approaches see refs. [2], [15]–[17].

Here, we present an approach to quantify selective reporting from large-scale datasets of published results. Such datasets are currently emerging in a number of research fields, including molecular medicine. Our approach extends previous work by Hedges and others [18]–[20] and uses weight functions to describe the chances of a study result to be published depending on its characteristics [10], [18]–[20]. Because the traces left due to selective reporting in the distribution of published results can be subtle, the statistical power for detecting biases based on weight function approaches is typically low. Therefore, a detailed modeling requires extensive datasets with preferably hundreds of studies. Such datasets can be generated by combining multiple datasets that are expected to be subject to the same bias. The approach presented here is specifically designed to quantify selective reporting from such combined datasets.

We use our approach to investigate data from the AlzGene database [21]. AlzGene is currently covering a set of over 1,200 case-control studies on nearly 2,300 different genetic markers for Alzheimer's disease (AD). A recent evaluation of these data demonstrated that there is a substantial excess of studies with statistically significant results, which strongly suggests the presence of selective reporting bias [22]. The genetic markers covered by the database might have different associations with the disease. For markers linked to the apolipoprotein E (APOE) gene, for example, there is strong evidence for an association. Other markers show nominally statistically significant associations in meta-analyses but their credibility is weak; and for most markers, there is likely no association with Alzheimer's disease [22].

Despite different association strengths, there is little reason to believe that fundamental differences exist between markers in how selective reporting bias is acting. Thus, even though selective reporting bias cannot be studied properly on a single marker, because most markers are only covered by a handful of studies each, the combined data are likely highly informative for quantitative models of selective reporting. Our approach exploits this and allows us to investigate whether initial publications are more biased than subsequent replications, and whether early replications are biased against initial results.

## Methods

### 1. Weight function approach

We build our approach on the selection model proposed by Hedges [19], [20]: results *X_1_,* …, *X_n_* are assumed to come from a normal distribution *X_i_ ∼ N(Δ, σ_i_^2^+σ^2^)* with a known within-study variance *σ_i_^2^*, an unknown between-study variance *σ^2^*, and an unknown mean effect size Δ. The relative chance for a result to be published is assumed to be depend on *Z_i_ = X_i_/σ_i_*, because the value of *Z_i_* typically determines the p-value associated with a study result. It is described by the weight function



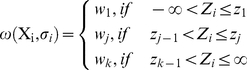
(1)


The weight function *ω(X_i_, σ_i_)* is stepwise constant, i.e. depending on *X_i_/σ_i_* it takes one of the values of *w =  (w_1_,* …, *w_k_)*. The function changes when *X_i_/σ_i_* crosses one of the interval boundaries *z =  (z_1_,* …, *z_k-1_)*. While the parameters *w =  (w_1_,* …, *w_k_)* are estimated from the data, the interval boundaries *z =  (z_1_,* …, *z_k-1_)* take fixed, pre-defined values. The discontinuities in the weight function at these pre-defined boundaries may be justified by the salience of p-values such as 0.05 in the current practice of interpreting study outcomes [19], [23]. Additionally, if a sufficiently large number of intervals are used, this weight function is flexible enough to reveal the actual shape of selection bias.

#### Density function and log likelihood

Applying the weight function to the probability density for the unbiased study results yields a weighted probability density given by
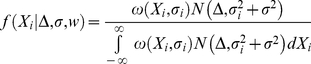
(2)


Because the weight function *ω(X_i_, σ_i_)* is stepwise constant, the integral in equation 2 can be rewritten as the sum
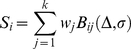
(3)


where *w =  (w_1_,* …, *w_k_)* denote the parameters of the weight function (eq. 1), and the terms *B_ij_* are given by
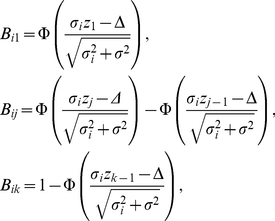
(4)


The log likelihood for the data *X =  (X_1_,* …, *X_n_)* is given by



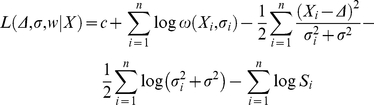
(5)


This function and first and second derivatives as given in [19] can be used to calculate maximum likelihood estimates for *Δ* and *σ^2^* in parallel with the parameters of the weight function, *w =  (w_1_,* …, *w_k_)*.

#### Extension to multiple datasets

The above approach simultaneously quantifies the parameters underlying the distribution of the unbiased data, and the parameters of the weight function *w =  (w_1_,* …, *w_k_)* describing selective reporting bias. We now assume that there are m datasets *X =  (X^(1)^,* …, *X^(m)^)* with *X^(i)^  =  (X_1_^(i)^,* … *X_n(i)_^(i)^)*. These datasets may differ in the underlying mean *Δ^(m)^* and between-study variance *σ^(m)2^*. However, all the datasets are assumed to be subject to the same selective reporting bias, i.e. the parameters *w =  (w_1_,* …, *w_k_)* are the same for all datasets. For the analysis of the AlzGene dataset, this means that we assume while the effect sizes and between-study variance might be different for different markers, selective reporting affects all markers in the same way. We therefore can simultaneously estimate *Δ =  (Δ^ (1)^,* …, *Δ^ (m)^)*, *σ =  (σ^(1)^,* …, *σ^(m)^)*, and *w =  (w_1_,* …, *w_k_)* by maximizing the log likelihood



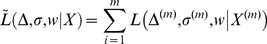
(6)


#### Multiple weight functions

**Figure 1:**
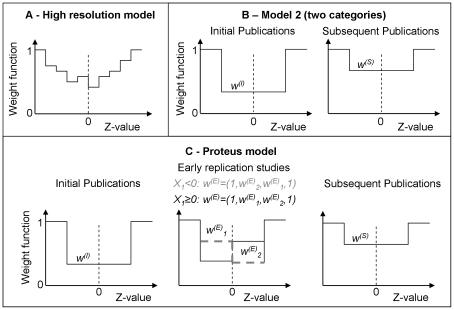
Weight functions for the publication bias models. The models specify the probability of a result to be published depending on the Z-value associated with the outcome. Weights are assumed to be stepwise constant, and are relative to the outer intervals where the weight function is set to one. (**A**) **High resolution model.** Using a stepwise constant weight function with many intervals allows studying the shape of publication bias. In our model we use 16 intervals. Model 1 is a simplified version with only three intervals, and a single free weight function parameter. (**B**) **Model 2 (two categories).** The weight function is assumed to differ for initial and subsequent studies. Initial studies with Z-values that fall into the mid interval are subject to weight *w^(I)^*, subsequent ones are subject to weight *w^(S)^*. Model 1 (see Methods), in contrast, uses a single one-parameter weight function that applies to all studies. (**C**) **Proteus model.** The weight functions are assumed to differ for initial studies, early replications, and subsequent publications. The shape for early replications depends on the result of the initial study (solid black line: initial result with *z≤0*, dashed grey line: initial result with *z>0*). This allows investigating whether early replication studies are more likely published if they oppose initial results. Model 3, described in detail in the Methods section, matches the Proteus model in complexity, but does not take into account that the sign of the initial study might have an impact on the weight function for early replications.

Moreover, if each study result *X_i_^(k)^* is associated with a categorical variable *K_i_^(k)^*, different weight functions can be used for the different categories. Instead of using a single weight function as in eq. 5 and 6, weight function *ω^(j)^* is used if *K_i_^(k)^* falls into category *j*. Using this approach we can, for example, use different categories for the initial studies, immediate follow-ups, and subsequent studies. This allows testing whether initial studies on a marker are more biased towards significant outcomes than late studies, or whether immediate replications tend to be biased against the result of the initial study. The use of multiple weight functions for quantifying the Proteus phenomenon is illustrated in Fig. 1.

#### Algorithm

Although estimating the parameters for these extensions requires somewhat more effort in handling the data, the likelihood function and its derivatives remain analogous to the ones given by Hedges [19], [20]. For maximizing the log likelihood function and determining a numerically approximated information matrix for calculation of the standard errors of the estimates, we use the BFGS algorithm as implemented in R. The approximated values are in line with results from simulation of datasets and subsequent re-estimation of the parameters. 95% confidence intervals (CI) are calculated as the estimate +/− 1.96 times the standard error. Details of the implementation are given in the Supporting Information S1.

#### Convergence

The unrestricted maximum likelihood approach used in our algorithm may not always converge. In our analysis, convergence problems arise for 22 of the 124 markers. To improve convergence, the between-study variance could be determined by non-iterative methods [24], but these approaches are difficult to combine with the maximum likelihood approach for estimating the weight function, and with model comparison based on the Akaike criterion. Alternatively, the between-study variance could be restricted to non-negative values. In our study, we exclude those markers that do not converge. In order to keep the different models comparable, we exclude the same markers in all the models. Re-estimation of parameters from simulated datasets similar to the one analyzed here indicates that these procedures do not lead to biases in the estimates. More sophisticated methods to deal with those markers that do not converge are subject of future research.

### 2. Data

#### Alzgene Data

The AlzGene database [21] contains data about the outcome of case-control studies on the association of genetic markers with Alzheimer's disease. The methodology for generating the AlzGene database is in detail described in ref. [21]. The data freeze used for this manuscript was performed on April 4th 2008 and includes 1,020 individual publications reporting association findings of 1,606 polymorphisms across 521 genetic loci. A total of 1,357 polymorphisms had non-overlapping genotype data available from at least one population. Note that this dataset is larger than that used in Kavvoura et al (2008; data freeze from January 31st 2007), which included 902 papers on 1,073 polymorphisms across 383 loci. For each study, a set of 2×3 tables is available that contains the numbers of homozygotes, heterozygotes and wild-types among AD cases and controls for each genetic marker analyzed in the study. These tables allow estimating a corresponding effect (log odds ratio) of a genetic marker on the probability of developing Alzheimer's disease. As in a previous review [22] of the data, we use per-allele odds ratios to estimate effect sizes. The log odds ratios *X_i_* and the corresponding standard errors *σ_i_* are estimated using a logistic regression. It should be noted that the p-values associated with observations in our model via the value of *Z_i_ = X_i_/σ_i_* are not necessarily identical to the ones given in the actual publications, where often additional factors such as age, gender or specific diagnosis (such as early-onset AD) might be included, and different statistical and genetic models might be used.

#### Criteria for including AlzGene data in our analysis

In the analysis presented here, we included results regardless of whether allele frequencies are in Hardy-Weinberg equilibrium. We included only markers with results from at least 4 independent publications, as judged by PubMed identifiers. In total, 124 markers fulfill this criterion. 22 markers are excluded because of convergence issues described above.

*Categories.* For modeling the Proteus phenomenon, we assigned one of three categories to each case-control study: initial findings, early replications, and late replications. Any studies on a specific marker that were published in the same year as the first case-control study on this marker fall into the first category (initial findings). Studies published within the next two years fall into the second category (early replications). All subsequently published studies fall into the third category (late replications). If a marker appears, for example, in publications from 2000, 2000, 2002, 2004, and 2005, the findings would fall into categories 1, 1, 2, 3 and 3, respectively. In an additional analysis we use PubMed IDs for an alternative categorization. Compared to using the publication year, PubMed IDs allow sorting papers at a finer temporal resolution. However, because particularly for publications from the 90's there is no strict relation between PubMed ID and date of publication, a PubMed ID based ranking is much less robust that a year-wise ranking. Results from the additional analysis are shown in the Supporting Information S1.

### 3. Model Specifications

**Figure 2:**
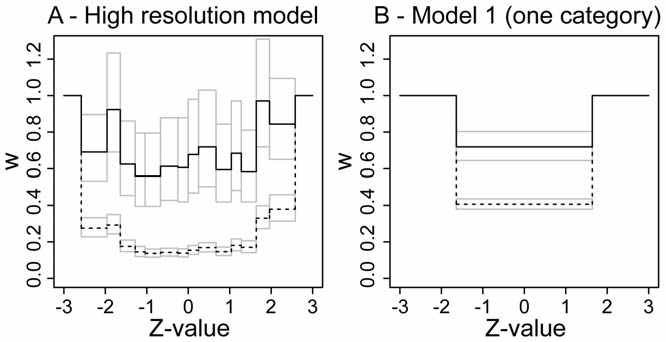
Results for the high-resolution models and the one category model (Model 1). Estimates for the high-resolution random-effects model are shown in solid lines, estimates for the fixed-effect model in dashed lines. Error bars show standard errors of the estimate. (**A**) **High resolution model.** For both the fixed-effect and random-effects model, the weights drop rapidly as *|Z|* decreases. At about *|Z| = 1.64*, the weights hit a bottom and remain relatively constant in the central intervals. This is what one would expect for publication bias: Non-significant results are subject to a similar bias, irrespective of the Z-value. (**B**) **Model 1.** In the random-effects model, bias in the mid interval is estimated as *w^(S)^ ≈ 0.7*. Under the fixed-effect model, we estimate *w^(S)^ ≈ 0.4*. The estimates for the bias in the fixed-effect model tend to be higher than in the random-effects model, because in the random-effects model, a high between-study variance offers an additional explanation for an excess of formally significant results.

To estimate selective reporting bias we use fixed-effect models and random-effects models (see Fig. 2). We use the following 6 models to characterize selective reporting bias. The models are illustrated in Figure 1.

#### Unbiased model

The weight function takes a constant value, *ω(X_i_, σ_i_)  = 1*, for all values of *X_i_* and *σ_i_*.

#### High-resolution model

We use 16 intervals with boundaries at *z =  (−2.58, −1.96, −1.64, −1.28, −1.04, −0.67, −0.25, 0, 0.25, 0.67, 1.04, 1.28, 1.64, 1.96, 2.58)*. The weight function is set to one for the outer intervals, i.e. *ω(X_i_, σ_i_)  = 1* for |*Z_i_*| *>2.58*. This model is used to obtain a picture of the shape of the weight function (Fig. 1A). The results were used to identify good choices for the boundaries for the following models with a reduced number of parameters.

#### Model 1 (one category)

We use a single weight function to model the data, i.e. do not use different categories for initial studies, early replications and late replications. The boundaries are chosen at *z =  (−1.64, 1.64)*. The weight function for this model, and for all following models, is set to one for the outer intervals, i.e. *ω(X_i_, σ_i_)  = 1* for |*Z_i_*| *>1.64*. The resulting model function has one parameter, *w^(S)^*, describing bias for all studies with *Z*-values that fall in the mid interval.

#### Model 2 (two categories)

This model is similar to Model 1, but uses two different weight functions. One weight function describes bias for the initial study, while the other weight function describes bias for all subsequent studies (Fig. 1B). The resulting model has two parameters, *w^(I)^* and *w^(S)^*, and allows detecting whether initial studies are more biased then subsequent ones.

#### Model 3 (three categories)

In this model, a third category is added to separately model early and late replication studies. The additional weight function is described by two additional parameters and uses boundaries at *z =  (−1.64, 0, −1.64)*. The model has four parameters: *w^(I)^* for initial studies that fall into the interval between *−*1.64 and 1.64, *w^(E)^_1_* for early replication studies with z-values between *−*1.64 and 0, *w^(E)^_2_* for early replication studies with z-values between 0 and 1.64, and *w^(S)^* for all subsequent studies that fall into the interval between *−*1.64 and 1.64. This model allows investigating whether early replication studies differ from later ones, and is designed to match the model for the Proteus phenomenon described in the next paragraph.

#### Proteus model

As in Model 3, we use three different weight functions, describing initial studies, early replications and late replications. The weight functions and parameters are analogous to the ones used in Model 3. However, the weight function for early replications is assumed to further depend on the outcome of the first study (see Fig. 1C). If the result falls into the interval between *−*1.64 and 1.64 and has the opposite sign of the initial study, the weight function is set to *w^(E)^_1_*. If it falls into the interval between *−*1.64 and 1.64 and has the same sign, weight *w^(E)^_2_* is used. If several studies on a marker are categorized as initial studies, we use the sign of the most extreme result. The Proteus model allows us to investigate whether subsequent studies that contradict the initial one are more likely to be published than studies that are in agreement with the initial one but do not show a strong effect. It has the same number of parameters as Model 3. A comparison between the two models is used to determine whether explicitly taking into account the sign of the initial study for modeling bias in early replications improves the model.

## Results

We find for the high-resolution model (Fig. 2A) that the estimated weights for results with standardized z-values (see Methods) between *−*1.64 and 1.64 are relatively constant. The weights start increasing as |*z*| increases over a value of 1.64. This is what one would qualitatively expect for selective reporting bias. Results that do not reach formal statistical significance are less likely published, irrespective of where exactly the result falls. The estimated weights in the 10 intervals covering the region between *−*1.64 and 1.64 fall roughly around *−*0.5 which corresponds to about 60% of non-significant results in any of these intervals being reported. For a fixed effects model, where the between-study variances are assumed to be zero (see Methods), the estimated weights are much lower, i.e. indicate an even stronger bias. This is because in the random-effects model there are two competing mechanisms for explaining a high abundance of extreme results: selective reporting bias and a high between-study variance. In the fixed-effect model, only the first mechanism is considered, leading to much stronger estimates of the bias.

The high-resolution model reveals an interesting shape of the weight function for results with z-values outside the interval between *−*1.64 and 1.64. Results with two-sided p-values between 0.05 and 0.01 (*1.96<|z|<2.58)* seem to be underrepresented when compared to results with a two-sided p-value smaller than 0.01 (|*z*| *>2.58*), and with two-sided p-values between 0.05 and 0.1 (*1.64<|z|<1.96*). This observation is somewhat surprising for classical publication bias where the *p = 0.05* value is considered to be a salient threshold for publication, but is in line with previous observations [22]. It may originate from using standardized rather than published p-values and is discussed in more detail further below.

Because the change of the weight function in the high-resolution model is highest at the boundaries of *z = −1.64* and *z = 1.64*, we used these boundaries in the subsequent models (Model 1–3 and Proteus model). Estimates for the simple random-effects one-parameter model (Model 1) are shown in Fig. 1B. The log weight function in the mid interval is estimated as *−*0.33 for the random effects model. This corresponds to a probability of reporting of about 72% (95% CI from 58% to 90%) relative to studies that fall into the outer intervals. This estimate is similar to the corresponding estimates in the high-resolution model, but note that we now use different outer intervals (i.e. boundaries at 1.64, and *−*1.64). The error in the estimate of the bias is much lower, because in the one-parameter model each interval contains data from several intervals of the high-resolution model (Fig. 2).

The results for the models designed to investigate the initial study bias and the Proteus phenomenon are summarized in Table 1. The estimates for Model 2 reveal that most of the bias stems from initial studies. These studies show a bias of log *w^(I)^  = −0.81*, suggesting that initial studies with standardized effects in the 1.64 to *−*1.64 interval have a chance of only 44% (95% CI from 32% to 63%) of being reported, relative to initial studies with results outside that interval. This bias is considerably stronger than the estimates for subsequent studies (*log w^(S)^  = −0.17*, respectively, corresponding to a probability of 84% compared with studies outside the interval; 95% CI from 66% to 107%). Thus, initial studies tend to be much more biased than subsequent ones. The potential origin and consequences of this effect are discussed further below. All estimates and their standard errors are summarized in Tab 1.

**Table 1:** Estimates of the weight function parameters.

	Random-effects model				
	Unbiased	Model 1	Model 2	Model 3	Proteus
***log w^(I)^***	-	-	*−*0.81 (0.17)	*−*0.82 (0.17)	*−*0.81 (0.17)
***log w^(E)^_1_***	-	-	-	*−*0.33 (0.17)	*−*0.11 (0.17)
***log w^(E)^_2_***	-	-	-	*−*0.24 (0.17)	*−*0.43 (0.17)
***log w^(S)^***	-	*−*0.33 (0.11)	*−*0.17 (0.12)	*−*0.08 (0.14)	*−*0.08 (0.14)
**Δ** ***_L_***	0	4.4	10.6	11.4	13.9
**Parameters**	0	1	2	4	4
**Δ_AIC_**	0	*−*6.8	*−*17.2	*−*14.8	*−*19.8

Standard errors of the estimates, calculated from a numerically approximated information matrix, are given in parentheses. Parameter *w^(I)^* described bias in initial studies, *w^(E)^_1_* and *w^(E)^_2_* describe bias in early replication studies in Model 3 and the Proteus model (Fig. 1 and Methods), and *w^(S)^* describes bias in subsequent studies. In Model 1, *w^(S)^* describes bias in all studies, in Model 2, all but initial studies. Further details are given in Fig. 1 and Methods. The AIC is given by *2k-2L*, where *L* is the maximized value of the log likelihood function and *k* is the number of parameters. The values given in the table are differences to the values for the unbiased model. Model 1 shows clear indication for selection bias. Model 2 shows that bias is larger for initial studies on a marker, compared to subsequent ones. The Proteus model indicates that non-significant studies opposing the initial result tend to be more likely published than non-significant studies confirming it. Note that the two parameters *log w^(E)^_1_* and *log w^(E)^_2_* are estimated relative to the outer intervals, and therefore the errors in the estimates are correlated. The difference between the two parameters is *log w^(E)^_1_ - log w^(E)^_2_ = *0.32. The standard error of the difference can be calculated from the variances and co-variances between the two estimates as determined by the information matrix and is given by *sqrt{var(log w^(E)^_1_)+var(log w^(E)^_2_) -2 covar (log w^(E)^_1_, log w^(E)^_2_)}  = *0.14. Thus non-significant studies confirming the initial result are published with a probability of 73% relative to non-significant studies opposing the initial result, with a confidence interval ranging from 55% to 96%. Model 3 shows that the direction of the second study does not matter per se. Selection bias for the early replication studies falls in between bias for initial and for subsequent results.

The results for the Proteus model show that studies that confirm the direction of the effect of an initial study but do not achieve formal statistical significance face more bias than studies with non-significant results that oppose the initial result in terms of the direction of the effect (chances are 73% relative to non-significant studies opposing the initial result, with a 95% CI from 55% to 96%; see Legend Table 1 for additional details). This is in line with observations described earlier [13], [14]. Model 3, which has the same number of parameters but does not make the weight function for the second study dependent on the sign of the first result, shows no differences between *w^(E)^_1_* and *w^(E)^_2_*, and yields a smaller change in the log likelihood score than the Proteus model. Based on the Akaike Information Criterion (AIC), the Proteus model would be favored for describing the given AD dataset. The single most important parameter in these models is the one to distinguish bias in initial publications from bias in subsequent ones, which indicates that the initial study bias is a very robust phenomenon.

The maximum likelihood approach outlined in the Methods section does not only yield estimates for the weight function parameters describing selection bias, but can also yield estimates for the association strength (log odds ratios) and between-study variances of each marker with AD after correcting for the selection pattern. Results for the unbiased random effects model (i.e. uncorrected estimates) and the Proteus model (i.e. estimates corrected for selective reporting) are given in the Supporting Information S2. A comparison of the result from these two models illustrates the effects of correction for selection bias on association strengths and between-study variances in the Proteus model. (A summary of the comparison is shown in the Supporting Information S1.) The differences between the estimates from the two models are rather subtle. The estimated absolute effect sizes in the Proteus model tend to be smaller than the estimated effect sizes from the uncorrected random effects model. However, standard errors for the estimates tend to be smaller, too, which means that the standardized effect sizes (z-values) remain similar. Moreover, the estimates of the between-study variances tend to be smaller for the Proteus models compared to the uncorrected random effects model. Without correcting for selection biases, effect sizes and between-study variances therefore tend to be overestimated.

## Discussion

Our results illustrate the strength that arises from combining datasets from a large number of primary studies within the same research field. The approach allows us to obtain a well-resolved picture of the weight function from a large body of literature (Fig. 2A). In line with previous results [22], our results from the high-resolution weight function point to a possible under-representation of results with standardized p-values between 0.05 and 0.01, and an excess of studies with p-values between 0.1 and 0.05. This particular shape of the weight function would have been difficult to obtain with other conventional weight functions such as exponentially declining, or s-shaped weight functions.

The observed over-representation of findings with standardized p-values between 0.1 and 0.05 might result from the use of standardized p-values in our analyses rather than the p-values given in the original publications. Often, when effect sizes and the corresponding p-values are presented in scientific publications, additional factors such as age, gender or specific diagnosis are included in the statistical analysis as covariates or to define subgroups, and the published results focus on the most promising subgroups. Because there is some flexibility in the design of the statistical model and analysis, and the analysis with the lowest p-values for the effect of interest is more likely to be presented in a publication, the p-values in the publications may often be lower than the standardized p-values. Therefore, many studies that achieve formal statistical significance at a level of 0.05 will have standardized p-values falling in the interval between 0.05 and 0.01. For the weight function, this may lead to an overrepresentation of studies with p-values from the interval between 0.05 and 0.1, and may imply that when standardized p-values are used, the largest discontinuity might not be expected at a p-value of 0.05 but at a higher level.

Most importantly, our results indicate that initial studies face a much stronger bias than subsequent ones. In line with previous observations, we find indication for the Proteus phenomenon: Early replications tend to be biased against the result of the initial publication. This effect, however, is smaller than the finding that initial findings are more biased than subsequent ones. Reasons and additional examples for initially inflated findings and for the Proteus phenomenon have been discussed earlier [8], [13], [14]. For evidence synthesis this is a severe problem, because when biases follow more complex patterns and differ for initial studies and subsequent ones, it is particularly difficult to correct for them. In order to correct for these biases one would have to examine the behavior of the whole field as data accumulate, and then assume that this behavior can be extrapolated to new data.

Our approach has potential methodological caveats. First, one important assumption for the weight function is the presence of discontinuities. Typically, these discontinuities are justified by the salience of particular p-values, such as 0.05, in the interpretation of research findings [19]. However, as illustrated by our high-resolution model, a sufficiently large number of published findings allows using a large number of intervals, and thus a stepwise constant weight function approach can give a better picture of the shape of the weight function than alternative approaches. Sensitivity to the normality assumption in the random-effects model is a potential caveat that has extensively been studied earlier [20]. While for our analysis, non-normality in the random-effects might influence the absolute estimates for the weights, it is implausible that it affects our findings regarding the presence of the Proteus phenomenon and initial study bias. Using weight functions that solely depend of the standardized effect sizes (z-value) is a further limitation, but additional variables can be easily implemented. In our analysis, this is illustrated by the use of different categories of results that are assumed to be affected by different biases. Distinctive categories could also be used when, for example, including data from genome-wide association studies where reporting may be by default more comprehensive. We therefore believe that the potential pitfalls associated with our methodology do not distort our results.

The approach outlined here can be easily adjusted to analyze selective reporting for a wide range of datasets. One could, for instance, analyze patterns of bias for the reporting of association between diseases and medical interventions or environmental factors. Unlike for gene-disease associations, the weight functions in these fields might not necessarily be symmetric, because protective effects of such factors might face a different bias than factors that increase risks. The timing of studies in other disciplines may also be slower, as compared to the rapid generation of results in genomics, and this may also affect the relative bias in replication results.

Moreover, the weight functions in our model can also be adjusted to capture the impact of further study-specific properties on bias. Biases might, for example, depend on the internal validity of a study. Reporting of design features that would allow an accurate assessment of the internal validity has not been optimal in the past genetic epidemiology literature (see [25]), but hopefully, with improved reporting in the future [26] such an analysis may be reliable enough to perform in future studies.

One might argue that, if reasons exist for not publishing all results that are obtained, a strong bias against non-significant outcomes specifically for early studies might be reasonable. Formally this can be assessed based on methods from information theory that allow quantifying the informativity of an experimental result [27]–[29]. Essentially, these methods allow quantifying how much an experimental observation changes our knowledge regarding a hypothesis. For some fields of research, including genetic associations, the prior chances of a randomly selected genetic variant to be associated with a disease is very low [30], [31]. In these fields, initial positive studies are typically more informative than initial negative studies. An initial finding that gene X is not associated with a particular disease offers little information, if no one expected it to be associated. If full publication comes with costs, there may be some benefit to not publish such a finding. However, if such a “negative” finding is not recorded anywhere even in brief, there may be a loss to other investigators who may continue spending time and effort on the same uninformative line of research. If selective reporting is seen as something inevitable, a detailed analysis of its prevalence, patterns, costs, and benefits is essential to understand its dynamics and how to handle it. Further analyses are required to determine whether there are forms of selective reporting that generate less severe problems in the context of evidence synthesis and may suggest how to optimize publication strategies under realistic costs and benefits.

## Supporting Information

Supporting Information S1 Supplementary information and additional results.
(DOC)

Supporting Information S2 Uncorrected and corrected estimates for the association strengths and between-study variances for the markers included in our analysis.
(CSV)
