# Supplementary material for: Quantifying Selective Reporting and the Proteus Phenomenon for Multiple Datasets with Similar Bias
Source: PLoS One. 2011 Mar 29;6(3):e18362. doi: 10.1371/journal.pone.0018362 (PMC3066227; doi:10.1371/journal.pone.0018362)
Supplement: Supporting Information S1 — Supplementary information and additional results. (DOC) [file pone.0018362.s001.doc]

*Supplementary Information for ms. “Quantifying Selective Reporting and the Proteus Phenomenon for Multiple Datasets with Similar Bias” by Thomas Pfeiffer, Lars Bertram and John P.A. Ioannidis*

*Implementation.* The algorithm is implemented in R, and uses the BFGS optimizer to determine the parameters that maximize the log likelihood function. We implemented two versions of the algorithm. In one version, all effect sizes, between-study variances and weight function parameters are estimated in parallel. This method becomes inefficient when data for many markers are fitted simultaneously. The second version exploits that the estimates for the effect sizes and between-study variances for the different markers depend on each other only via the estimate of the weight function parameters. The log likelihood function can therefore be more efficiently maximized by iterating two steps. First, maximum likelihood estimates for all effect sizes and between-study variances are determined for given weight function parameters. Second, maximum likelihood estimates of the weight function parameters are determined for the new effect sizes and between-study variances. These steps are iterated until convergence is achieved. In our study we use the second version of the algorithm to get an initial estimate. The first version of the algorithm is subsequently used for “polishing” the optimum and determining the information matrix for calculation of the standard errors of the maximum likelihood estimates. An implementation of our algorithm will be made available on the internet together with a sample input file and the corresponding output.

*Effect sizes and between-study variances of the individual markers.* A table containing the estimates for effect sizes and between-study variances of the markers included in this study is provided in the Supplementary Dataset <SuppData.csv>. The file contains estimates for the unbiased random effects model and the Proteus model. A summary is shown in Table 2 of the Supplementary Information. The comparison of the two models shows that the estimated effect sizes in the Proteus model, which are corrected for selective reporting, are smaller than the estimated effect sizes for the unbiased model. The estimated standard errors are smaller, too, such that the standardized z-values remain similar. The between-study variances tend to be smaller for the Proteus model.

*Additional analysis based on PubMed ID categorization.* For an additional analysis we used PubMed IDs to refine the ordering of the results for their time of publication. Note that PubMed IDs are not assigned based on publication date. Therefore the relation between ID and date of publication is not perfect. We thus first sort the publications by year, and use the PubMed IDs to identify the order of publications within each year.

Based on the resulting ranking we use two different assignments for the categories “initial publication”, “early replication”, and “late replication”. For both analyses, the category “initial publication” is used only for the first publication, i.e. the one that has the lowest PubMed ID within the first year at which a marker appears in a published case-control study. For the first analysis (Assignment 1), the category “early replication” is assigned only to the second-ranked publication, while all subsequent publications are categorized as “late replication”. This is an extreme categorization that allows testing whether the Proteus phenomenon is driven by publications following immediately after the initial publication.

For our second analysis (Assignment 2), we use a categorization similar to the one used in the main manuscript. Results that are published within the year of the initial publication or the next two year are categorized as “early replication”, the remaining results as “late replication”. Note that the alternative categorization does not affect Model 1 because in this model, publication bias is estimated over all publications. For Model 2, both assignments give the same results, because the way initial publications are assigned is the same. The results are shown in the Table 2 of the Supplementary Information. Differences in AIC and likelihood score are relative to the unbiased model, as in Table 1 of the manuscript.

Using PubMed IDs to identify initial studies increases the estimated bias for initial publications. The estimated probability for a non-significant initial study decreases to about 34% (CI from 23% to 50%). Both models show substantial differences in the estimates for the bias among early replications. In Assignment 1, we observe a stronger bias and no Proteus effect for this category. Errors for the estimates are larger, because much fewer results are categorized as early replications. In Assignment 2, we observe a similar bias as for the year-wise analysis (Table 2), and evidence for the Proteus phenomenon. These results suggest that those publications that follow immediately after the initial publication are similar to initial publications. In part this might also result from mis-categorizations due to the non-chronological assignment of PubMed IDs. The Proteus phenomenon does not necessarily appear immediately after the initial publication, but is consistently observed over the next few years. The Proteus Model in Assignment 2 achieves the highest AIC score among all models tested.

| ***Model*** | **Absolute association strength (log odd ratios)** | | **Standard errors of log odd ratios** | | **Between-study variances** | |
| --- | --- | --- | --- | --- | --- | --- |
|  | Median | Mean | Median | Mean | Median | Mean |
| ***Uncorrected*** | 0.069 | 0.092 | 0.067 | 0.085 | 0.014 | 0.031 |
| ***Proteus*** | 0.057 | 0.081 | 0.062 | 0.074 | 0.010 | 0.023 |

**Supplementary Information - Table 1. Comparison between uncorrected and corrected estimates of association strength and between-study variances.** In addition to the estimates for the weight function, the Proteus model also yields maximum likelihood estimates for the association strength of a marker with AD (log odds ratios) and the between-study variances. These estimates are implicitly corrected for the estimated selection bias. Comparison with maximum likelihood estimates from a random effects model that does include selection bias illustrates the systematic effects of the correction. The individual estimates for each marker and each model are given in the Supplementary Dataset (SuppData.csv), and are here summarized by the median and mean values of the absolute association strength, their standard errors, and the between study variances. Note that the summary is based on all 102 markers entering our analysis. Only for a few of these markers there is substantial evidence of an association with AD. The estimated absolute effect sizes in the Proteus model tend to be smaller than the estimated effect sizes from the uncorrected random effects model. Standard errors for the estimates tend to be smaller, too. The standardized effect sizes (z-values) and the corresponding p-values therefore remain approximately the same. The estimates of the between-study variances tend to be smaller for the Proteus models compared to the uncorrected random effects model. Thus without correcting for selection biases, effect sizes and between-study variances tend to be overestimated.

|  | **Random-effects model** | | | | |
| --- | --- | --- | --- | --- | --- |
|  |  | **Assignment 1** | | **Assignment 2** | |
|  | **Model 2** | **Model 3** | **Proteus** | **Model 3** | **Proteus** |
| ***log w^(I)^*** | -1.07 (0.19) | -1.07 (0.19) | -1.07 (0.19) | -1.09 (0.20) | -1.07 (0.19) |
| ***log w^(E)^_1_*** | - | -0.71 (0.32) | -0.58 (0.31) | -0.30 (0.15) | -0.15 (0.16) |
| ***log w^(E)^_2_*** | - | -0.45 (0.30) | -0.56 (0.30) | -0.28 (0.16) | -0.38 (0.15) |
| ***log w^(S)^*** | -0.19 (0.12) | -0.15 (0.12) | -0.15 (0.12) | -0.09 (0.14) | -0.09 (0.14) |
| ***∆_L_*** | 14.2 | 15.8 | 15.3 | 14.9 | 16.7 |
| **Parameters** | 2 | 4 | 4 | 4 | 4 |
| **∆_AIC_** | -24.4 | -23.6 | -22.6 | -21.8 | -25.4 |

**Supplementary Information - Table 2.** **Estimates for the additional analyses.** The notation is analogous to Table 1 in the manuscript. In Assignment 1, where only the first publication (as judged by the PubMed ID) is categorized as “initial study”, and only the second publication as “early replication”, there is good evidence for an initial study bias, but no evidence for the Proteus phenomenon. In Assignment 2, where all papers appearing within two years after the initial study are categorized as “early replication”, we observe both the initial study bias and the Proteus phenomenon. The Proteus Model in Assignment 2 has the best AIC score. This suggests that the Proteus phenomenon does not necessarily appear immediately after the initial publication but is operating over the next few years.
